# A direct, real-time, spectrophotometric assay for measuring ENPP1-catalyzed cGAMP hydrolysis

**DOI:** 10.1016/j.jbc.2026.113078

**Published:** 2026-04-27

**Authors:** Marisa M. Michalchik, Zane Lombardo, Demetrios T. Braddock, Wenxiang Cao, Enrique M. De La Cruz

**Affiliations:** 1 Department of Molecular Biophysics and Biochemistry, Yale University, New Haven, Connecticut, USA; 2 Department of Pathology, Yale University School of Medicine, New Haven, Connecticut, USA

**Keywords:** ENPP1, 2′3′-cGAMP, cGAMP hydrolase, enzyme kinetics, enzymology, spectrophotometry

## Abstract

The ectonucleotidase ENPP1 is the major extracellular hydrolase of the innate immune system activator 2′3′-cyclic GMP-AMP (cGAMP). Tumors that overexpress ENPP1 and rapidly degrade cGAMP avoid immune surveillance in the tumor microenvironment and are highly resistant to cancer immunotherapy. Inhibition of cGAMP degradation by ENPP1 has emerged as a promising strategy to improve cancer therapies. A direct, real-time assay of ENPP1 enzymatic activity would benefit quantitative evaluation of candidate ENPP1 inhibitors. The nonphysiological substrate p-nitrophenyl 5′-thymidine monophosphate is commonly used for this purpose, as it offers a readily detectable colorimetric readout that can be evaluated in real time. However, compounds that potently inhibit p-nitrophenyl 5′-thymidine monophosphate hydrolysis can weakly inhibit ENPP1 with physiological nucleotide substrates, highlighting the importance of testing ENPP1 inhibitors with native substrates (*i.e.*, cGAMP). No direct, real-time assays for cGAMP hydrolysis are established. Here, we present a real-time, spectrophotometric assay to monitor ENPP1-catalyzed cGAMP hydrolysis. The increase in extinction coefficient associated with conversion of substrate(s) to AMP and GMP products is used to convert time courses of absorbance change to rates of product formation. Time courses of GMP product formation generated from the absorbance change superimpose with those generated by the direct measurement of GMP product concentration *via* chemical quench-flow and HPLC analysis. ENPP1 inhibition by the nonhydrolyzable ATP analog, α,β-methylene-ATP, yields an inhibition constant (*K*_I_) comparable to the independently determined binding affinity. This spectroscopic assay can be performed using a standard, laboratory UV-vis spectrophotometer and has the potential to be scaled up to a high-throughput, multiwell plate setup.

The STING pathway agonist 2′3′-cyclic GMP-AMP (cGAMP) is critical for activation of the nonspecific innate immune response, a necessary first line of defense culminating in a more specific immune response by the adaptive immune system. Several types of cancer evade immunosurveillance by overexpressing the major cGAMP hydrolase, ectonucleotide pyrophosphatase phosphodiesterase (ENPP1), on their cell surface. ENPP1 rapidly degrades extracellular cGAMP, essentially shutting down the innate immune response and the downstream adaptive immune system activation. Tumors with high ENPP1 expression levels have lower levels of T cell infiltration and are more resistant to both traditional cancer treatments and immunotherapy (1).

Inhibition of cGAMP hydrolysis by ENPP1 has emerged as a promising cancer immunotherapy strategy to maintain high cGAMP concentrations in the tumor microenvironment, thereby enhancing the natural antitumor response and improving the efficacy of traditional cancer treatments. Small molecule inhibition of ENPP1 has shown promising results in treating many cancers in animal models, and several inhibitors are being evaluated in human clinical trials (2, 3, 4, 5). Quantitative knowledge of ENPP1 enzymatic activity is necessary for assessing inhibitor potency and the mechanism of cGAMP hydrolysis inhibition.

A convenient, real-time enzymatic assay for ENPP1-catalyzed cGAMP hydrolysis is not available. The most common assay for ENPP1 enzymatic activity is a colorimetric assay using the nonphysiological ENPP1 substrate p-nitrophenyl 5′-thymidine monophosphate (pNP-TMP; *e.g.* (6)), which yields an absorbance change upon liberation of the hydrolysis products. pNP-TMP can be a reliable proxy for gauging inhibitory capabilities of drug candidates, but the inhibition of ENPP1 enzymatic activity does not always translate to physiological substrates (7, 8). For example, potent inhibitors of pNP-TMP hydrolysis by ENPP1 have had modest effects with physiologically relevant substrates, including nucleotide and non-nucleotide inhibitors that act competitively or noncompetitively/uncompetitively (7, 8, 9). This difference highlights the need to evaluate candidate ENPP1 inhibitors with native substrates, including cGAMP. ENPP1-catalyzed hydrolysis of cGAMP is often assayed using radio-labeled substrate and/or chromatographic separation involving multiple steps and standard curves for quantification (4, 10, 11, 12). A coupled luminescent assay has also been developed (13), but this requires the resulting AMP to be converted to ATP in a series of enzymatic reactions before the amount of ATP is quantified using a luciferase-luciferin reaction. Thus, the evaluation and characterization of ENPP1 inhibitors would benefit from a direct and convenient, real-time assay of ENPP1 enzymatic activity.

Here, we present a real-time spectrophotometric assay to monitor ENPP1-catalyzed cGAMP hydrolysis. Our analysis with both cGAMP and pApG (3′–5′ adenylyl-guanosine dinucleotide) substrates confirms that time courses of GMP product production generated using this method superimpose with those generated through direct measurement of GMP product concentration using chemical quench-flow and HPLC analysis. This assay can be successfully performed by hand mixing with a standard laboratory absorbance spectrophotometer and has the potential to be scaled up to a multiwell plate setup for high-throughput screening of potential ENPPI inhibitors.

## Results

### Determination of the extinction coefficients for cGAMP and pApG

**Figure 1:**
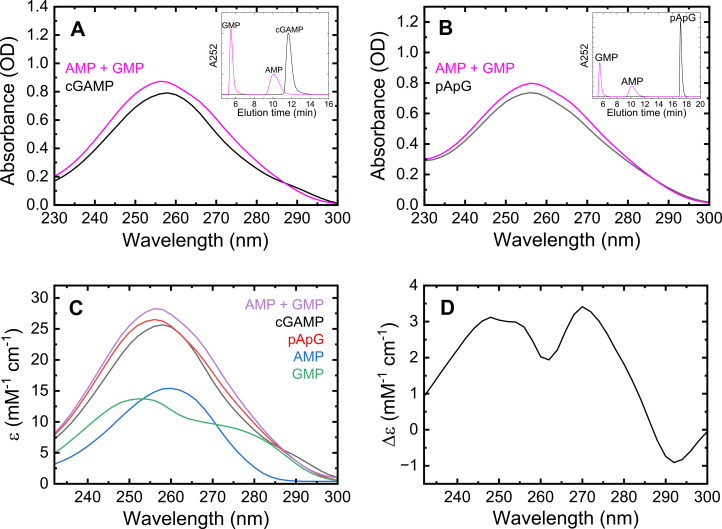
**Extinction coefficient determination for cGAMP substrate, pApG intermediate, and AMP + GMP products.***A*, absorbance spectra of a cGAMP solution (100 μM) before (*black* line) and after (*magenta* line) complete digestion by ENPP1. Inset shows HPLC chromatograms of the same cGAMP samples. *B*, absorbance spectra of a pApG solution (100 μM) before (*black* line) and after (*magenta* line) complete digestion by ENPP1. Inset shows HPLC chromatograms of the same pApG samples. *C*, extinction coefficient as a function of wavelength (232–300 nm) for all nucleotide substrates, intermediates, and products: cGAMP substrate (*black*), pApG linear intermediate (*red*), AMP product (*blue*), GMP product (*green*), and the sum spectrum of AMP and GMP products (*purple*). *D*, difference in extinction coefficient between equal concentrations of products (AMP + GMP) and substrate (cGAMP) at all wavelengths observed (232–300 nm).

Degradation of both cGAMP and the linear dinucleotide pApG to their mononucleotide products (AMP and GMP; confirmed by HPLC; Fig. 1, *A* and *B* inset) is accompanied by a change in absorbance (Fig. 1, *A* and *B*) and thus a change in the overall sum of extinction coefficients (Fig. 1, *C* and *D*). Using the known extinction coefficients for AMP and GMP products, we calculate (Equation 2) extinction coefficient values for cGAMP and pApG at their peak wavelengths (258 and 256 nm, respectively) of 25.7 and 26.5 mM^−1^ cm^−1^, respectively under our solution conditions (pH 7.4; Table 1). The extinction coefficients of nucleotides and dinucleotides depend on the solution conditions (*e.g.*, salt concentrations and pH), but the extinction coefficients of AMP and GMP change very little (∼1%) between pH 7.0 and 7.5.

**Table 1:** Extinction coefficients for nucleotides used in this study

Extinction coefficient	cGAMP	pApG	AMP	GMP	AMP + GMP
λ_max_ max (nm)	258	256	259	252	256
ε_max_ (mM^−1^ cm^−1^)	25.7	26.5	15.4	13.7	28.3
ε_260_ (mM^−1^ cm^−1^)	25.3	25.5	15.4	11.9	27.3
ε_AMP + GMP260_ - ε_substrate260_ (mM^−1^ cm^−1^)	2.0	1.8	-	-	-

### Absorbance detection assay for ENPP1-catalyzed cGAMP and pApG hydrolysis

**Figure 2:**
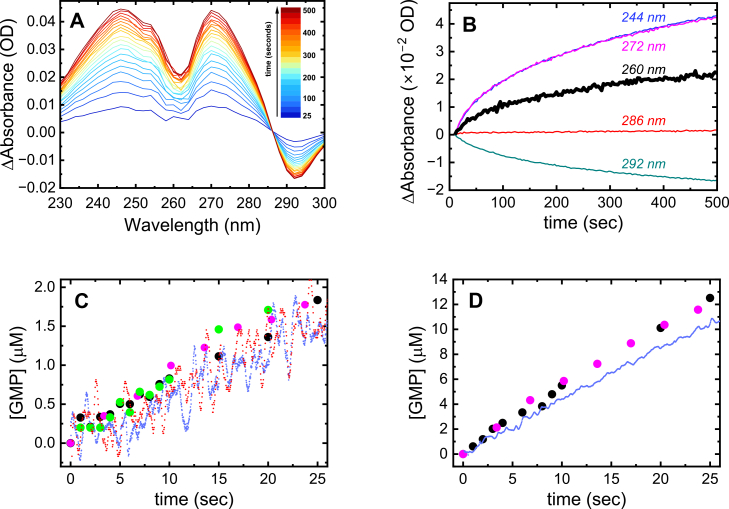
**ENPP1-catalyzed cGAMP and pApG hydrolysis can be monitored in real-time by absorbance change at 260 nm.***A*, time resolved difference spectra (*A*_*t*_-*A*_*0*_) showing the absorbance change at every wavelength upon mixing ENPP1 (400 nM) with 100 μM cGAMP. *B*, time courses of absorbance change upon mixing ENPP1 (400 nM) with 100 μM cGAMP at different wavelengths: two, where the largest changes in absorbance are observed (244 nm and 272 nm), the isosbestic point (286 nm), and the wavelength with the largest decrease in absorbance (292 nm). The *black* line indicates our chosen wavelength of 260 nm, where *ε*_cGAMP_ ≈ *ε*_pApG_, for monitoring product formation. *C*, time courses of GMP accumulation after mixing ENPP1 (50 nM) with cGAMP (100 μM) measured directly by quench-flow and HPLC quantification of GMP in solution (*black circles* for ENPP1 and *green circles* for ENP-HM102 or absorbance change at 260 nm (*small blue circles* – stopped-flow w/ENPP1; *small red circles* - stopped-flow with ENP-HM102; *magenta circles* – UV-vis spectrometer with ENPP1). Assays were performed independently. *D*, time courses of GMP accumulation after mixing ENPP1 (100 nM) with pApG (50 μM) measured directly by quench-flow and HPLC quantification of GMP in solution (*black circles*) and absorbance change at 260 nm (*blue line* – stopped-flow with ENPP1; *magenta circles* – UV-vis spectrometer with ENPP1). Assays were performed independently.

The absorbance change associated with the degradation of either cGAMP or pApG can be utilized to measure real-time product liberation during ENPP1-catalyzed hydrolysis of these dinucleotide substrates. Because we know the extinction coefficients of the reaction substrates (*ε*_cGAMP_ or *ε*_pApG_) and products (*ε*_AMP_ and *ε*_GMP_), we can readily convert the absorbance change at any given time point (*A*_t_ - *A*_0_, where *A*_t_ is the absorbance at time *t* and *A*_0_ is the initial absorbance) to the concentration of generated product (Equations (3), (4), (5), (6), (7)). The magnitude and direction of the absorbance change vary with the detection wavelength (Fig. 2, *A* and *B*).

The substrate pApG is also an intermediate product generated during ENPP1-catalyzed hydrolysis of cGAMP (14). Absorbance detection at 260 nm, where cGAMP and pApG have nearly identical extinction coefficients (ε_cGAMP_ ≈ ε_pApG_, 25.3 and 25.5 mM^−1^ cm^−1^, resp.; Fig. 1*C*), selectively monitors the production of the final mononucleotide products and avoids contributions of the pApG intermediate to the absorbance signal. We therefore monitor absorbance change at a wavelength of 260 nm (Fig. 2*B*) to ensure that the observed absorbance change reflects the liberation of the final mononucleotide products (AMP and GMP). The change in extinction coefficient when cGAMP is completely hydrolyzed into AMP and GMP products is 2.0 mM^−1^ cm^−1^ at 260 nm (Table 1), resulting in an ∼8% change in the measured absorbance. Monitoring at alternate wavelengths like 242 nm and 272 nm yields improved signal to noise ratios due to larger absorbance changes, but the signals include contributions from the pApG intermediate (Fig. 2*B*).

### Determination of steady-state kinetic parameters for ENPP1-catalyzed cGAMP and pApG hydrolysis

Time courses of GMP production during cGAMP and pApG hydrolysis measured both by direct quantification of nucleotide product (chemical quench-flow) and absorbance change (hand mixing in a UV-vis spectrometer) are superimposable (Fig. 2, *C* and *D*); the data collected by rapid mixing using a stopped-flow instrument deviated slightly over 25 s, but the difference in the rate of absorbance change determined from the slope of the best fit to a linear function was ∼10%. The nearly identical behavior demonstrates the validity of the absorbance signal change to monitor cGAMP and pApG hydrolysis. This approach can thus be used to determine steady-state kinetic parameters for cGAMP and pApG substrate hydrolysis from the [substrate]-dependence of the initial velocity (*v*_0_).

**Figure 3:**
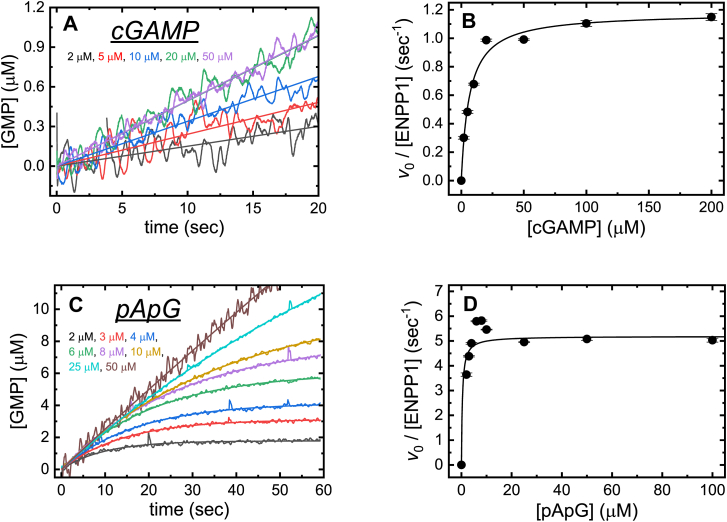
**Steady state catalysis of cGAMP and pApG hydrolysis by ENPP1.***A*, time courses of GMP accumulation after mixing ENPP1 (50 nM) with a range of [cGAMP] (2–50 μM). Each time course shown is an average of three traces. The continuous lines through the data points represent the best fit to a linear function. *B*, [cGAMP]-dependence of the initial rate of product liberation (*v*_0_), obtained from fits in (*A*), per enzyme active site. Uncertainty bars fall within the data points and represent the standard errors in fitting to time courses in (*A*). The continuous line through the data represents the best fit to the Briggs-Haldane equation (Eq. 10), yielding a *K*_M,cGAMP_ value of 6.5 ± 1.2 μM and a *k*_cat, cGAMP_ value of 1.2 ± 0.1 s^−1^ for ENPP1-catalyzed cGAMP hydrolysis. *C*, time courses of GMP accumulation after mixing ENPP1 (50 nM) with a range of [pApG] (2–50 μM). Each time course shown is an average of three traces. The 2 to 25 μM data is collected with a 1 cm pathlength cell and the 50 μM data is collected with a 0.2 cm pathlength cell. The continuous lines through the data points represent the best fit to Equation 9. *D*, [pApG]-dependence of the initial rate of product liberation (*v*_0_), obtained from fits in (*C*), per enzyme active site. Uncertainty bars fall within the data points and represent the standard errors in fitting to time courses in (*C*). The continuous lines through the data represent the best fit to the Briggs-Haldane equation (Eq. 10), yielding a *K*_M,pApG_ value of 0.4 ± 0.3 μM and a *k*_cat, pApG_ value of 5.2 ± 0.3 s^−1^ for ENPP1-catalyzed pApG hydrolysis. Uncertainties in the *k*_cat_ and *K*_M_ values originate from standard errors in the best fits of the data (*B*) and (*D*) to Equation 10.

Time courses of steady state cGAMP and pApG hydrolysis are initially linear (Fig. 3, *A* and *C*) but became nonlinear over longer time scales (Figs. 2*B* and 3*C*) due to substrate depletion and product inhibition (15). The *v*_0_ can be determined from the initial slope. Alternatively, the entire time courses can be analyzed following methods developed for determining *v*_0_ from nonlinear steady-state enzyme kinetic time courses (16).

**Figure 4:**
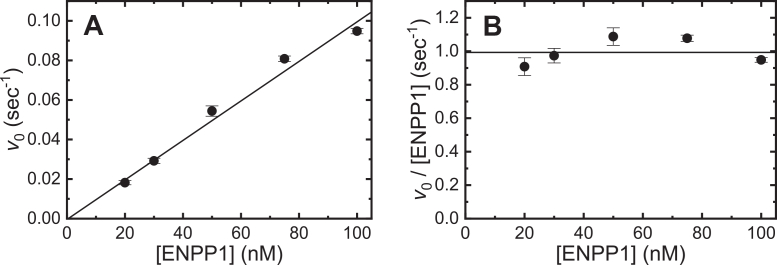
**[ENPP1]-dependence of cGAMP hydrolysis.***A*, [ENPP1]-dependence of the initial velocity (*v*_0_) of cGAMP hydrolysis. The continuous line represents the best fit to a linear function yielding a *v*_0_/[ENPP1] at 100 μM cGAMP of 1.0 ± 0.1 s^−1^. *B*, [ENPP1]-dependence of the initial cGAMP hydrolysis velocity per ENPP1 active site (*v*_0_/[ENPP1]) The continuous line represents the best fit to a linear function yielding a *v*_0_/[ENPP1] at 100 μM cGAMP of 1.0 ± 0.1 s^−1^ with a slope of ∼ 0. Uncertainty bars represent the standard errors from best fits of the time courses. Uncertainties in the *v*_0_/[ENPP1 values represent standard errors in the linear fits of the data, as shown in (*A*) and (*B*).

The *v*_0_ of ENPP1-catalyzed cGAMP or pApG hydrolysis depend hyperbolically on the initial [cGAMP] (Fig. 3*B*) or [pApG] (Fig. 3*D*). The maximum turnover rate per ENPP1 at saturating [cGAMP] substrate (*k*_cat, cGAMP_) is 1.2 ± 0.1 s^−1^ with a Michaelis constant (*K*_M,cGAMP_) of 6.5 ± 1.2 μM, yielding a *k*_cat_,_cGAMP_/*K*_M,cGAMP_ (“specificity constant”; (17)) of 0.2 ± 0.04 μM^−1^ s^−1^ under our solution conditions (Fig. 3, *A* and *B*, Table 2). These steady-state kinetic parameters are independent of the [ENPP1] over the range examined (Fig. 4, *A* and *B*) and are comparable to those reported by others using radio-labeled cGAMP, with hydrolysis measured by thin-layer chromatography under different solution conditions (*k*_cat, cGAMP_ = 0.76 s^−1^, *K*_M,cGAMP_ = 12.1 μM, and *k*_cat, cGAMP_/*K*_M,cGAMP_ = 0.06 μM^−1^ s^−1^; (4)). Steady-state hydrolysis of pApG by ENPP1 is more efficient, with a maximum enzyme turnover rate per ENPP1 at a saturating [pApG] substrate (*k*_cat, pApG_) of 5.2 ± 0.3 s^−1^ and a *K*_M,pApG_ of 0.4 ± 0.3 μM, yielding a *k*_cat, pApG_/*K*_M,pApG_ value of 13 ± 10 μM^−1^ s^−1^ (Fig. 3, *C* and *D*, Table 2).

**Table 2:** Steady-state kinetic parameters for ENPP1-catalyzed cGAMP and pApG hydrolysis

Parameter	Value
*k*_cat, cGAMP_	1.2 (±0.1) sec^−1^
*K*_M,cGAMP_	6.5 (±1.2) μM
*k*_cat, cGAMP_/*K*_M,cGAMP_	0.2 (±0.04^a^) μM^−1^ sec^−1^
*k*_cat, pApG_	5.2 (±0.3) sec^−1^
*K*_M,pApG_	0.4 (±0.3) μM
*k*_cat, pApG_/*K*_M,pApG_	13 (10^a^) μM^−1^ sec^−1^

a Uncertainties in *k*_cat_/*K*_M_ values were calculated according to error propagation from the errors of *k*_cat_ and *K*_M_.

### Small-molecule inhibition of ENPP1-catalyzed cGAMP hydrolysis

**Figure 5:**
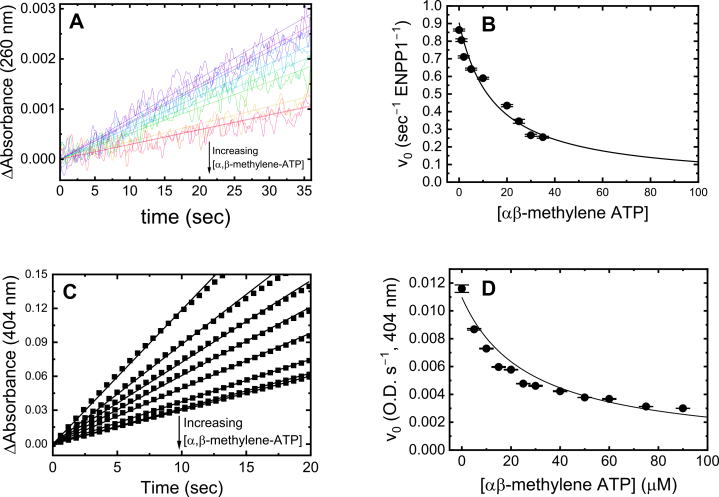
**α,β-methylene-ATP inhibits ENPP1 hydrolysis of cGAMP and pNP-TMP.***A*, time courses of cGAMP hydrolysis by ENPP1 with increasing [α,β-methylene-ATP] (50 nM ENPP1 and 20 μM pNP-TMP). Each time course shown is an average of three traces. *B*, inhibition of ENPP1 by [α,β-methylene-ATP] with cGAMP substrate. Continuous lines through the data represent the best fits to a function for competitive inhibition (Equation 1). Uncertainty bars represent the standard errors in fitting the time courses in (*A*). The resulting inhibition constants (*K*_I_) is 3.6 ± 0.3 μM. *C*, time courses of pNP-TMP hydrolysis by ENPP1 with increasing [α,β-methylene-ATP] (50 nM ENPP1 and 70 μM pNP-TMP). Each time course shown is an average of three traces. *D*, inhibition of ENPP1 by [α,β-methylene-ATP] with pNP-TMP substrate. Continuous lines through the data represent the best fit to a function for competitive inhibition (Equation 1). Uncertainty bars represent the standard errors in fitting the time courses in (*C*). The resulting inhibition constants (*K*_I_) is 2.3 ± 0.1 μM. Uncertainties for *K*_I_ values originate from standard errors in fits of data in (*B)* and (*D*).

This absorbance-based assay can also be used to characterize inhibitors of ENPP1-catalyzed cGAMP hydrolysis. We demonstrate this through use of the nonhydrolyzable ATP analog, α,β-methylene-ATP, which acts as a competitive inhibitor of ENPP1 (7, 8, 15). The rate of ENPP1-catalyzed cGAMP hydrolysis decreases with the [α,β-methylene-ATP] (Fig. 5*A*); similar behavior was observed with pNP-TMP (Fig. 5*C*). The *v*_0_ determined from these cGAMP and pNP-TMP hydrolysis time courses depend hyperbolically on the [α,β-methylene-ATP] (Fig. 5, *B* and *D*). The inhibition constants (*K*_I_) are determined from the best fits of the data to a hyperbola in the form of the competitive inhibition equation (Equation 1):

(1) $$\begin{array}{c} v_{0}=\frac{V_{\max}\left[S\right]}{K_{M}\left(1+\frac{\left[I\right]}{K_{I}}\right)+\left[S\right]} \end{array}$$

where *K*_M_ is the Michaelis constant for cGAMP (6.5 μM, Fig. 3*B*) or pNP-TMP (6.6 μM, Fig. S1) substrates (*S*), and *I* is the [α,β-methylene-ATP]. The *K*_I_ values are 3.6 ± 0.3 μM and 2.3 ± 0.1 μM with cGAMP and pNP-TMP substrates, respectively. These *K*_I_ values for α,β-methylene-ATP are comparable to *K*_I_ values previously determined for using pNP-TMP (3.3 μM; (7, 8)) and the ∼1 μM binding affinity of ENPP1 for α,β -methylene-ATP (15). We note that the range of [α,β-methylene-ATP] that could be monitored by absorbance at 260 nm was limited because it also absorbs at the detection wavelength.

## Discussion

### Real-time spectrophotometric assay for the quantification of cGAMP hydrolysis

Here, we developed an absorbance-based assay to directly monitor ENPP1-catalyzed cGAMP hydrolysis into AMP and GMP in real time utilizing a standard laboratory spectrophotometer. Time courses of AMP and GMP formation measured by HPLC superimpose with those measured by absorbance, demonstrating the validity of the direct detection method and approach. The assay can reliably quantitate the potency of ENPP1 enzymatic activity inhibition by small molecule ligands, as demonstrated with the nonhydrolyzable ATP analog, α,β-methylene-ATP. The enzymatic activity of any cGAMP hydrolase (*e.g.*, ENPP3 and SMPDL3B) can be monitored, with potential for scaling up to a multi-well plate format for high-throughput screening.

We emphasize that a consistent absorbance change of a given sample can be reliably monitored as a function of time despite the small absolute absorbance changes. Measuring absolute absorbance changes over certain time intervals between different samples is challenging due to uncertainties and errors introduced in sample preparation (*e.g.*, pipetting variability and errors). Accordingly, we do not recommend this assay for comparing the absolute absorbance values with single-point assays (*i.e.*, one reaction time point) of different samples and suggest that it be used only for analysis of time courses, which reliably monitor AMP and GMP production of a given sample.

### Steady-state kinetics of ENPP1-catalyzed dinucleotide hydrolysis

ENPP1 catalyzes the degradation of cGAMP with a maximum turnover rate (*k*_cat, cGAMP_) of 1.2 ± 0.1 s^−1^ and a *K*_M,cGAMP_ of 6.5 ± 1.2 μM (Fig. 3*B*). These values are within a factor of two of one report in the literature (3) determined using radio-labeled cGAMP, with hydrolysis measured by thin-layer chromatography under different solution conditions (100 mM Tris (pH 9 or pH 7.5), 150 mM NaCl, 500 μM CaCl_2_, 10 μM ZnCl_2_) but differ significantly from other values, also determined under different assay conditions than used here. Li *et al.* (4) reported a *k*_cat, cGAMP_ of 4 s^−1^ and a *K*_M,cGAMP_ of 15 μM at a different pH and solution salt conditions (0.2% (v/v) NP-40, 20 mM Tris–HCl (pH 9.0), 2 mM CaCl_2_, 200 μM ZnCl_2_), but unspecified temperature. Similarly, Namasivayam *et al.* (10) reported a *k*_cat, cGAMP_ of 5.36 ± 0.32 s^−1^ and a *K*_M,cGAMP_ of 32.6 ± 6.5 μM for the ENPP1 cGAMP hydrolase using a single time point assay and different solution conditions and temperature (37 °C in a buffer containing 1 mM MgCl_2_, 2 mM CaCl_2_, 10 mM Ches buffer (pH 9.0)). The differences in temperature, pH, and salt could potentially account for these discrepancies.

ENPP1 catalyzes the degradation of the cGAMP hydrolysis intermediate product and substrate, pApG, with a maximum enzyme turnover rate per ENPP1 at a saturating [pApG] substrate (*k*_cat, pApG_) of 5.2 ± 0.3 s^−1^ and a *K*_M,pApG_ of 0.4 ± 0.3 μM, yielding a *k*_cat, pApG_/*K*_M,pApG_ value of 13 ± 10 μM^−1^ s^−1^ (Fig. 3, *C* and *D*, Table 2). Thus, pApG is a more efficient, or specific, ENPP1 substrate than cGAMP, but not ATP (*k*_cat,ATP_/*K*_M,ATP_ = 50 μM^−1^ s^−1^; (15)). Importantly, the >4-fold more rapid *k*_cat_ with pApG substrate compared to cGAMP indicates that at least one step before pApG hydrolysis limits the overall hydrolysis cycle of cGAMP conversion to AMP and GMP.

## Experimental procedures

### Protein and reagents

All reagents were the highest purity commercially available. Glycosylated human ENPP1 ((18) and construct 770 in ref. (19), consisting of the human ENPP7 signal sequence for export, the somatomedin B 1&2, catalytic, and endonuclease domains of human ENPP1, followed by the human IgG1 Fc domain; referred to as ENPP1 throughout) was purified from CHO cells as described (19), dialyzed against PBS_plus_ buffer (1X PBS buffer pH 7.4, 11 μM ZnCl_2_, 20 μM CaCl_2_), and stored at −80 °C. Commercially produced recombinant ENPP1 fragment (referred to as ENP-HM102 in the text) consisting of residues Lys98-Asp925 purified from HEK293 cells was purchased from KACTUS Bio for the comparison to ENPP1 (Fig. 2*C*) and yielded identical results. All experimental measurements were carried out at 25 °C in ENPP1 assay buffer designed to mimic salt concentrations in blood (20 mM Tris–HCl (pH 7.4), 154 mM NaCl, 0.014 mM ZnCl_2_, 1 mM MgCl_2_, 1 mM CaCl_2_, 4.5 mM KCl). The ENPP1 and nucleotide concentrations stated are final after mixing, unless specified otherwise.

### Determination of extinction coefficients and conversion factors for concentration changes

HPLC analysis indicates cGAMP and its linear intermediate product, pApG, are both fully hydrolyzed to AMP and GMP by ENPP1 (Fig. 1, *A* and *B* insets). This hydrolysis is accompanied by a change in absorbance and therefore overall extinction coefficients. Using the known extinction coefficients for AMP and GMP products, extinction coefficients for both cGAMP and pApG substrates can be precisely determined.

Extinction coefficients for AMP and GMP at wavelengths other than their peak absorbance wavelengths (15,400 M^−1^ cm^−1^ at 259 nm for AMP and 13,700 M^−1^ cm^−1^ for GMP at 252 nm) were calculated using the ratio of absorbance at the alternate wavelength to the absorbance at peak wavelengths. Extinction coefficients for cGAMP and pApG at different wavelengths were determined as previously described for Ap3A and Ap4A (20, 21) using the following equation:

(2) $$\begin{array}{c} \varepsilon_{substrate}=\frac{A_{substrate}*\;\varepsilon_{AMP+GMP}}{A_{AMP+GMP}} \end{array}$$

where [AMP + GMP] = [substrate] and the path length is identical.

Using the nucleotide extinction coefficient (*ε*) values (Table 1), time (*t*)-dependent changes in absorbance (*A*) can be converted to rates of substrate depletion and product formation using the following formula:

(3) $$\begin{array}{c} \frac{\frac{\Delta A}{\Delta t}}{\left(\varepsilon_{AMP}+\varepsilon_{GMP}\right)-\varepsilon_{substrate}} \end{array}$$

where ΔA/Δt is the time-dependent change in absorbance (in units of OD s^−1^) normalized to a 1 cm path length. The total absorbance (*A*_*t*_) at any time point during cGAMP hydrolysis (using a 1 cm path length) is given by:

(4) $$\begin{array}{c} A_{t}=\left[cGAMP\right]\times \varepsilon_{cGAMP}+\left[pApG\right]\times \varepsilon_{pApG}+\left[AMP+GMP\right]\times \varepsilon_{AMP+GMP} \end{array}$$

If *ε*_cGAMP_ = *ε*_pApG_, as is the case at 260 nm (Table 1), then the total absorbance is given by:

(5) $$\begin{array}{c} A_{t}=\left(\left[cGAMP\right]+\left[pApG\right]\right)\times \varepsilon_{cGAMP}+\left[AMP+GMP\right]\times \varepsilon_{AMP+GMP} \end{array}$$

(6) $$\begin{array}{c} A_{t}=\left({\left[cGAMP\right]}_{initial}-\left[AMP+GMP\right]\right)\times \varepsilon_{cGAMP}+\left[AMP+GMP\right]\times \varepsilon_{AMP+GMP} \end{array}$$

The concentrations of AMP and GMP products generated from cGAMP substrate hydrolysis at any time point can then be calculated from:

(7) $$\begin{array}{c} \left[AMP+GMP\right]=\frac{{A_{t}-}_{(}\times {\left[cGAMP\right]}_{initial})}{\varepsilon_{AMP+GMP\;}-\varepsilon_{cGAMP}} \end{array}$$

Similarly, the concentrations of AMP and GMP products generated from pApG substrate hydrolysis can be calculated from:

(8) $$\begin{array}{c} \left[AMP+GMP\right]=\frac{{A_{t}-}_{(}\times {\left[pApG\right]}_{initial})}{\varepsilon_{AMP+GMP\;}-\varepsilon_{pApG}} \end{array}$$

### Steady-state kinetics of cGAMP and pApG hydrolysis

Time courses of ENPP1-catalyzed cGAMP and pApG hydrolysis were measured with 50 nM ENPP1 and a range (2–200 μM) of [substrate]. Enzyme concentration was varied where indicated in the text (Fig. 4). Initial velocities (*v*_0_) were determined by fitting time courses of GMP product accumulation or absorbance change to either a linear function, over applicable, initial time ranges, or by fitting the entire nonlinear time courses to the following equation developed for determining *v*_0_ from nonlinear steady-state enzyme kinetic time courses (16):

(9) $$\begin{array}{c} \left[P\right]=\frac{v_{0}}{\eta }\left(1-e^{-\eta t}\right) \end{array}$$

where *v*_0_ is the initial velocity of AMP product production, *η* is the decay constant of the steady-state rate (*i.e.*, the first derivative of the turnover velocity) from substrate depletion and/or product inhibition, and *t* is time. The inhibition of ENPP1 by the AMP product (14, 15) occurs earlier in time courses collected at higher ENPP1 concentrations, causing the data to deviate from linearity almost immediately. To avoid this issue, we suggest performing this assay with a concentration of ENPP1 between 20 and 100 nM, a range across which the initial velocity is linearly dependent on the ENPP1 concentration (Fig. 4, *A* and *B*). Lowering the ENPP1 concentrations is also an option, provided time courses are monitored over longer time scales that allow for adequate absorbance changes to occur.

The Michaelis constants for cGAMP and pApG (*K*_M,cGAMP_ and *K*_M,pApG_, respectively) substrates (*S*) and the maximum ENPP1 turnover rate constants (*k*_cat,cGA_ and *k*_cat, pApG_) were obtained by fitting the [substrate]-dependence of the initial velocity (*v*_0_) to a rectangular hyperbola in the form of the familiar Briggs-Haldane equation:

(10) $$\begin{array}{c} v_{0}=\frac{k_{cat,S}\left[ENPP1\right]\left[S\right]}{\left[S\right]+K_{M,S}} \end{array}$$

### Direct measurement of chemical cleavage of cGAMP and pApG

Chemical cleavage of cGAMP and pApG was measured by rapidly mixing ENPP1 and substrate using a KinTek RQF-3 Chemical-Quench-Flow apparatus, quenching with 3 M formic acid at the indicated times and subsequently quantifying nucleotide species in solution by HPLC analysis (15). The reverse phase HPLC program for nucleotide analysis consists of an initial 1 min of sample loading with buffer A (20 mM potassium phosphate, pH 6.0) at a flow rate of 0.1 mL min^−1^, followed by 10 min of washing with buffer A, 10 min of gradient with 0 to 100% buffer B (20 mM potassium phosphate, pH 6.0, 20% (v/v) methanol), and 10 min of column re-equilibrating for the next sample with buffer A at a flow rate of 1 mL min^−1^. Nucleotides were detected by UV absorption at 252 nm. Peak identities in absorption chromatograms are determined by nucleotide standards in the same reaction buffer and quenching reagent, under the same running conditions as reaction samples. The nucleotide quantification is carried out by converting the integration of the corresponding UV absorption peak to nucleotide concentration according to a standard integrated peak area versus concentration curve.

### Stopped-flow absorbance change assay

Time courses of absorbance change at 260 nm were recorded after rapidly mixing ENPP1 with nucleotide (cGAMP or pApG) using an Applied Photophysics SX.20 MV-R stopped-flow apparatus, monitoring conversion of dinucleotide substrate to AMP and GMP products. Observation cell pathlengths of 10 mm and 2 mm were used to collect time courses using nucleotide concentrations less than or greater than 30 μM, respectively. Time courses were collected for 40 to 60 s with 0.02 s time resolution. Time courses presented are averages of at least three traces. Due to tight product binding (15, 22, 23), product inhibition occurs early in the reaction, which can lead to nonlinearity in the time courses. It is, therefore, recommended to limit the [ENPP1] to < 100 nM. Equation 9 can be used to fit entire nonlinear time courses, or linear fits can be restricted to the initial velocity regions (∼20 s for [ENPP1] = 5 to 100 nM).

### UV-vis spectrophotometer absorbance change assay

Data was also collected using a Hewlett Packard 8452A Diode Array Spectrophotometer, allowing for the collection of absorbance spectra with a time resolution of ∼3 s. The collection of full spectra allowed for the generation of time courses across a range of wavelengths. Cuvettes with pathlengths of 10 mm and 3 mm were used to collect time courses using cGAMP concentrations less than or greater than 30 μM, respectively. Reactions were initiated by adding 130 μl of cGAMP to a cuvette containing 20 μl of ENPP1. Since product inhibition of ENPP1 occurs quickly, the cGAMP solution was added to a cuvette already placed in the spectrophotometer while it was actively acquiring spectra to get as close to true time zero as possible. Steady-state pNP-TMP hydrolysis was measured by absorbance at 404 nm (19, 24).

## Data Availability

All data are presented in the manuscript figures and/or Online Supplementary Material. Raw data and analysis done will be shared upon request by emailing the corresponding author.

## Supporting information

This article contains supporting information.

## Conflicts of interest

D. T. B. is an inventor on patents owned by Yale University for therapeutics treating ENPP1 deficiency and receives research and consulting support from Biomarin. All other authors declare that they have no conflicts of interest with the contents of this article.
