# Supplementary material for: A direct, real-time, spectrophotometric assay for measuring ENPP1-catalyzed cGAMP hydrolysis
Source: J Biol Chem. 2026 Apr 27;302(6):113078. doi: 10.1016/j.jbc.2026.113078 (PMC13223937; doi:10.1016/j.jbc.2026.113078)
Supplement: Supplementary Figure Legends [file mmc1.docx]

**Supplementary Figure Legends**

**Figure S1. [pNP-TMP]-dependence of the observed steady-state hydrolysis rate of ENPP1**. **A)** Time courses of ENPP1 hydrolysis of pNP-TMP (10 nM ENPP1 with varying [pNP-TMP]). The continuous lines through the data represent fits to equation 9. Deviation from linearity results from substrate depletion at low [pNP-TMP]. Each time course shown is an average of three traces. **B**) [pNP-TMP]-dependence of the observed steady-state hydrolysis rate of ENPP1. The continuous line through the data represents the best fit to a rectangular hyperbola (Eq. 10) yielding a *K*_M_ for pNP-TMP of 6.6 ± 0.7 µM from the [pNP-TMP] at half-maximum velocity. Uncertainty bars represent the standard errors in fitting the time courses in S1A. Uncertainties for *K*_M_ values originate from standard errors in fits.
