# Supplementary figures and images for: A direct, real-time, spectrophotometric assay for measuring ENPP1-catalyzed cGAMP hydrolysis
Source: J Biol Chem. 2026 Apr 27;302(6):113078. doi: 10.1016/j.jbc.2026.113078 (PMC13223937; doi:10.1016/j.jbc.2026.113078)

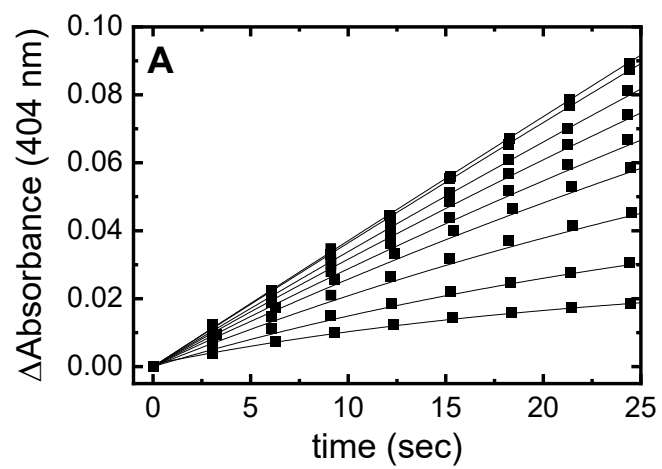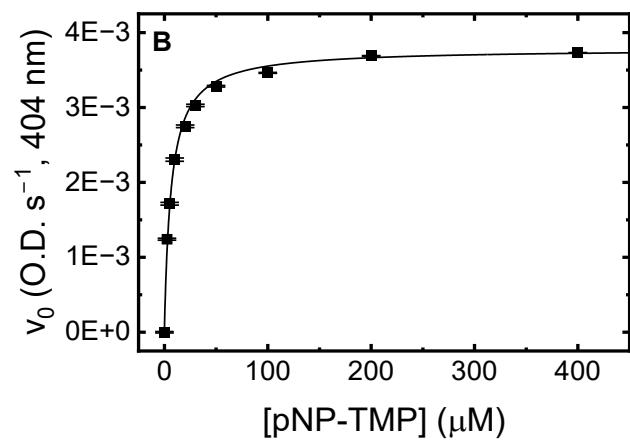

Supplement: Supplementary Figure S1 [file mmc2.pdf]
